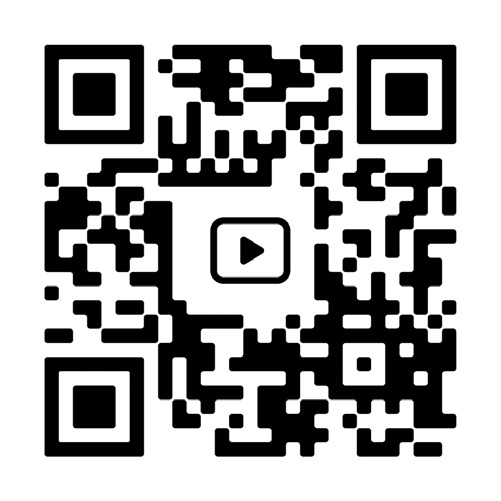# Bioengineering strategies of the uterus towards improving current investigative models and female reproductive health

**Published:** 2019-03

**Authors:** H Campo, I Cervelló, A Pellicer

**Affiliations:** Fundación IVI, Instituto de Investigación Sanitaria La Fe, Valencia, Spain;; Instituto Valenciano de Infertilidad (IVI), Rome, Italy; Instituto de Investigación Sanitaria La Fe, Valencia, Spain.

**Keywords:** Tissue engineering, decellularization, uterine bioengineering, ECM hydrogel, embryo culture

## Abstract

Ever since the inception of artificial reproductive technologies (ART), new advances have been developed in the lab and translated to the clinic to improve the reproductive outcome of patients. Tissue engineering (TE) adopts ideas and concepts from biology, bioengineering and material science amongst others, resulting in a promising and burgeoning multidisciplinary field of investigation within regenerative medicine.

The main objective of the work presented in this thesis was to use TE based approaches to create different types of natural biomaterials obtained from decellularized porcine or rabbit uteri. We investigated if these different bioscaffolds could improve current investigative in vitro models while also showing potential to be used as therapeutic solutions. Decellularized whole organs are acellular vascularized scaffolds that could be used to create tissue-engineered, transplantable organs. However, they can also be processed further into thin sections, ECM hydrogels and coatings, and were used as biocompatible tissue-specific substrates for cell and embryo culture. Two animal models were used, the pig model demonstrated that our perfusion-based protocol (with or without a freeze/thaw step) successfully decellularizes large uteri, yielding a biocompatible material. Secondly, we adapted this protocol for the rabbit uterus and converted the acellular endometrium into tissue-specific ECM hydrogels and coatings. After characterization of these substrates their effect on in vitro embryo development was also examined. While DC organs could one day be used to resolve the main issues plaguing transplantations, endometrial ECM sections, hydrogels and coatings have shown the potential to become a platform used in the culture of stem/progenitor cells and primary culture cells to better maintain their tissue-specific phenotype, improving in vitro models. Furthermore, ECM hydrogels could possibly be used in the future in vivo, as part of a treatment of Asherman’s syndrome and endometrial atrophy.

## Introduction

The original aim of Tissue Engineering (TE), as defined by Langer and Vacanti, is to restore, maintain, or improve function of tissues or whole organs (regenerative medicine, in vivo application) (Langer and Vacanti, 1993). However, these engineered tissues have also evolved into a viable option for drug testing, disease modeling, and precision medicine (In vitro applications) (Wobma and Vunjak-Novakovic, 2016). What sets TE apart is the use of biomaterials or scaffolds that serve as a physical environment that supports cells; for this they must possess several important characteristics. This includes the correct mechanical properties such as rigidity and elasticity but they also need to provide bioactive cues to regulate the cellular activity, to able to sequester growth factors and lastly to be degradable (Chan and Leong, 2008).

These scaffolds can be synthetic or from natural origins, but it goes without saying that the ideal scaffold is the original extracellular matrix (ECM). The ECM was once thought to act mainly as structural support (Bornstein et al., 1977), but now it is clear that this complex mixture of fibrous structural proteins, specialized proteins, GAGs and proteoglycans (PGs) plays an important role in cell migration, proliferation and differentiation (Beattie et al., 2009; Turner and Badylak, 2013). Furthermore, for every tissue and organ there is a bidirectional interchange between the cell and its surrounding ECM called “dynamic reciprocity”. This also means that the dynamic composition and three-dimensional organization of the ECM is unique and for each tissue, in order for them to preform tissue-specific cellular functions during homeostasis and injury (Nelson and Bissell, 2006; Schultz et al., 2011; Thorne et al., 2015). This is also the case for the uterus and endometrium, which goes through cyclical, scar-free regeneration throughout the menstrual cycle, these changes are also reflected in its ECM composition (Hawkins and Matzuk, 2008; Evans et al., 2011).

Decellularization (DC) is an innovative method to create complex natural scaffolds. Here, all cellular material is removed from a tissue or organ, while maintaining the hierarchical complexity, composition and the three-dimensional (3D) ultrastructure of the extracellular matrix (ECM) (Gilbert et al., 2006). By removing the cellular antigens, detrimental immunological or inflammatory reactions are attenuated. This means that biomaterials from both allogeneic and xenogeneic origins can be used. Interestingly, in some in vitro studies using xenogeneic ECM yielded better results than their allogeneic counterpart (Young and Goloman, 2013). The last and unique advantage is the ability to decellularize entire organs by perfusion. By using the vascular system, decellularization agents are delivered to all layers of the organ, where the cells are lysed. Cellular debris is then removed, leaving an acellular vascularized three-dimensional bioscaffold. In the last years this TE technique has become more popular, also in reproductive medicine (Amorim, 2017), various protocols have been studied to efficiently decellularize uterine tissues (Table I). 

**Table I:** — Decellularization protocols of the uterus and uterine tissues.

Tissue	Decellularization protocol	Reference
Rat and Human myometrial segments	Immersion in 70% ethanol for 24hr, H_2_O for 1hr and for 3 or 24hrs in Trypsin (0.25% in 1X EDTA).	(Young and Goloman 2013)
Rat uterine segments	Immersion for 1hr in SDS (0.1% in PBS), 1hr in SDS (1% in PBS), 2hrs in SDS (1% in PBS), 24hrs in Triton X- 100 (1% in PBS), 24hrs in Triton X- 100 (3% in PBS), 48hrs in Triton X- 100 (3% in PBS) or treated by HHP: 10 min at 980MPa (10°C or 30°C). All were washed for 1 week at 4°C in 0.9% NaCl, 0.05 M MgCl2·6H2O, 0.2 mg/ml DNAse I and 1% P/S	(Santoso et al. 2014)
Whole rat uterus	Perfusion with SDS (0.01%, 4°C) for 24hrs, SDS (0.1%, 4°C) for 24hs, SDS (1%, RT) for 24hrs, 15min with H2Od, 30min with Triton X-100 (1%) and washed extensively with sterile PBS.	(Miyazaki and Maruyama 2014)
Whole rat uterus	Perfusion using 3 protocols: Protocol 1 (P1, 5 cycles): 4hrs DMSO (4% in PBS+A), 4hrs Triton X-100 (1% in PBS+A), 30 min PBS+A (+ stored O/N). Protocol 2 (P2): identical to P1 but dilutions made in dH2O + sodium azide (0.05%; H2Od+A), F/T cycle between cycle 2 and 3. Protocol 3 (P3, 5 cycles): 6hrs SDC (2% in H2Od+A), 2hrs H2Od+A, stored O/N in H2Od+A at RT.	(Hellström et al. 2014)
Whole rat uterus	Perfusion protocols P1, P2 and P3 as described above.	(Hellström et al. 2016)
Mice uterine segments	Immersion for 1hr in SDS (1% in PBS) and washed for 1 week at 4°C in a 0.2 mg/ml DNAse I solution (0.9% NaCl, 0.05 M MgCl2·6H2O and 1% P/S)	(Hiraoka et al. 2016)
Human endometrial segments	Immersion for 48hrs 0.25% Triton X-100 + 0.25%SDC (37°C), 4 days DMEM/F12 (4°C), 24hrs 100 μg/ml ribonuclease and 150 IU/ml DNase I (37°C), 24hrs DMEM/F12 (4°C).	(Olalekan et al. 2017)
Human endometrial segments	Immersion in 0.25% Triton X-100 + 0.25 %SDC for 48hrs (37°C), DMEM/F12 (4°C) for 4 days, 100 μg/ml ribonucle-ase and 150 IU/ml DNase I (37°C) for 24hrs and for 24hrs in DMEM/F12 (4°C).	(Xiao et al. 2017)
Fragmented bovine uterine horn	Immersion for 2-4 weeks in SDS (0,1%, 4°C).	(Jakus et al. 2017)

Abbreviations: DMSO: Dimethyl sulfoxide; F/T: freeze/thaw; HPP: High hydrostatic pressure; PBS: phosphate buffered saline; P/S: penicillin/streptomycin; H2Od: distilled water; SDS: sodium dodecyl sulfate.

This complex acellular tissue can be processed in various ways to serve different purposes (Agmon and Christman, 2016). For instance, sections or blocks of decellularized tissues can de be made and seeded with different types of cells to create three-dimensional cell culture systems (Baptista et al., 2011; Campo et al., 2017). Lastly, decellularized scaffolds can be also converted into ECM hydrogels or coatings. These injectable ECM hydrogels undergo a non-toxic, collagen-based self-assembly process into a nanofibrous hydrogel when incubated at 37°C or introduced in vivo, which makes them an interesting option for regenerative medicine purposes and especially for minimally invasive procedures. By converting the tissues into substrata such as hydrogels or coatings, the spatial organization of the ECM proteins is lost, but important specific biochemical elements remain intact. These biological cues are contained in a more flexible substrate expanding its in vivo and in vitro usability.

When looking at the in vitro studies, it is demonstrated that this type of substrates not negatively affects the viability of cell lines (Wolf et al., 2012), primary culture cells (Lee et al., 2014) and stem cells (DeQuach et al., 2010; Zhang and Dong, 2015). The best example for the therapeutic potential of this type of hydrogels is a porcine-derived myocardial ECM hydrogel, which is currently in clinical trials to treat patients after myocardial infarction (MI) (ClinicalTrials.gov Identifier: NCT02305602). Rat and porcine MI models showed an improvement of neovascularization, cardiac function and increase in cardiac muscle. It was also demonstrated that the porcine-based hydrogels performed similarly to human myocardial pre-gel solutions and even displayed more reliable gelation in vivo (Johnson et al., 2014).

**Figure 1:**
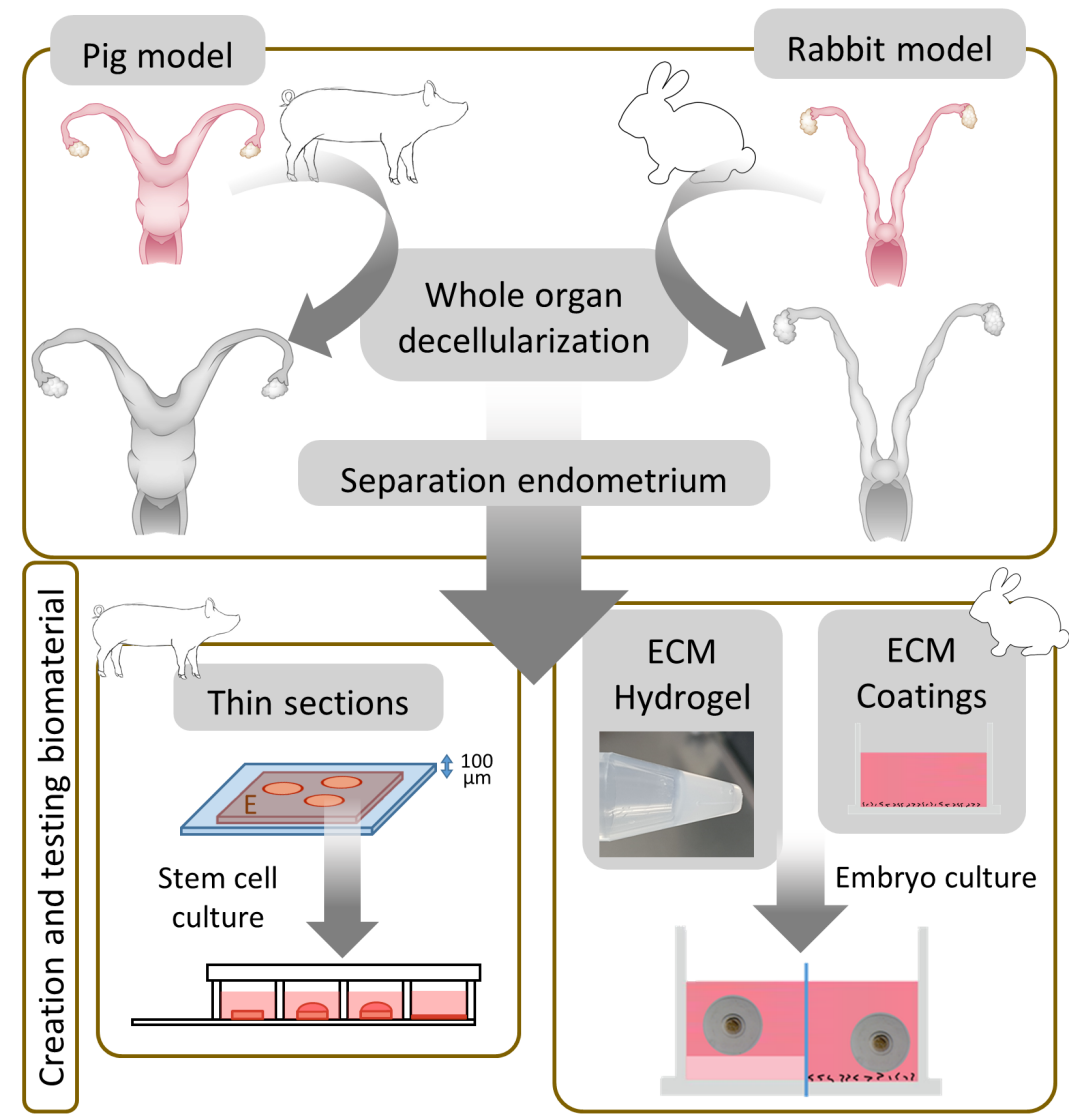
Overview of tissue engineered biomaterials created, characterized and tested. In a first phase, whole porcine and rabbit uteri are decellularized, followed by the separation of the endome-trium and the processing of this tissue into 100-micron thin sections, ECM hydrogels and ECM coat-ings. Biological in vitro tests demonstrated their usability for future in vivo applications.

For the purposes of this thesis, two animal models were used (Figure 1). First, we developed a perfusion-based protocol usable for large pig uteri, the effect of a prior freeze/thaw step was also assessed. DC efficiency and the effect on the extracellular matrix (ECM) was tested thoroughly by histology techniques, protein and DNA quantification, vascular corrosion cast, immunofluorescence, scanning and transmission electron microscopy. Finally, in vitro biocompatibility was tested by recellularizing endometrial sections using human endometrial Side Population stem cell lines. In the second part of the thesis, we intended to corroborate if the differences in the cyclically and drastically changing endometrium are translated to tissue-specific ECM hydrogels and coatings, possibly affecting the development of embryo culture in a rabbit model.

## Objectives

The main objective of this study is to develop tissue-engineering approaches based on decellularized uterine tissues obtained from whole organs to improve reproductive medicine. Within this goal five specific objectives were defined:

- To establish a protocol for the perfusion-based decellularization of large reproductive organs, namely the porcine and rabbit uterus.
- To test the biocompatibility of the newly created bioscaffolds by first recellularizing acellular pig endometrial disks with human Side Population stem cells and creating organoid-like structures.
- To develop a microdissection-based protocol to separate the rabbit endometrium from whole decellularized organs and convert it into a tissue-specific ECM hydrogel and coating.
- To assess the usability of these ECM hydrogels and coatings for the in vitro culture of rabbit embryos.
- To investigate the effects of substrata from decellularized synchronous and non-synchronous endometrial tissues in the embryo culture model.

## Materials and methods

### 1. The pig model: decellularization of whole uterus and recellularization of the acellular endometrium

#### 1.1 Whole uterus decellularization

The goal of the pig model was to develop a perfusion-based DC protocol usable for large reproductive organs, resulting in a biocompatible bioscaffold. The effect of a prior freeze/thaw (F/T) step was also assessed.

In total six uteri were selected based on their morphology and much attention was given to their vascular system, three uteri were frozen/thawed (protocol 1: F/T), and three uteri were decellularized immediately (protocol 2: fresh, F). The uterine artery of a single horn was cannulated, leaving the other one without perfusion. This was carefully attached to a peristaltic pump and an initial perfusion of PBS for one hour was done to remove the remaining blood and cell debris. The perfusion speed for all protocols was set at a physiological flow rate of 15 mL/min (Geisler et al., 2012). The complete protocol is detailed in Table II.

**Table II:** — Basis protocol for perfusion-based decellularization of large uteri.

	Reagent	Concentration	Duration
	PBS	1X	1 hr
Cycle 1	SDS	0,1%	18 hrs
	H_2_Od	-	30 min
	Triton-X100	1%	30 min
	PBS	1X	5 hrs
Cycle 2	SDS	0,1%	18 hrs
	H_2_Od	-	30 min
	Triton X-100	1%	1 hr
	PBS	1X	5 hrs

The cyclical basis protocol was first established for the pig uterus, to assess the effect of previously freezing the organ 2 protocols were compared: Frozen/Thawed (F/ T) and Fresh (F). Slight modifications were made for the rabbit uterus: the 5 hr PBS step in the second cycle was replaced by 1 hr of 1X PBS followed with a 2μg/mL DNase 1 solution, diluted in 1,3 mM MgSO4 and 2 mM CaCl2 step for one hour and lastly 3 hrs of 1X PBS. Abbreviations: PBS: phosphate buffered saline; H2Od: distilled water; SDS: sodium dodecyl sulfate.

**Figure 2:**
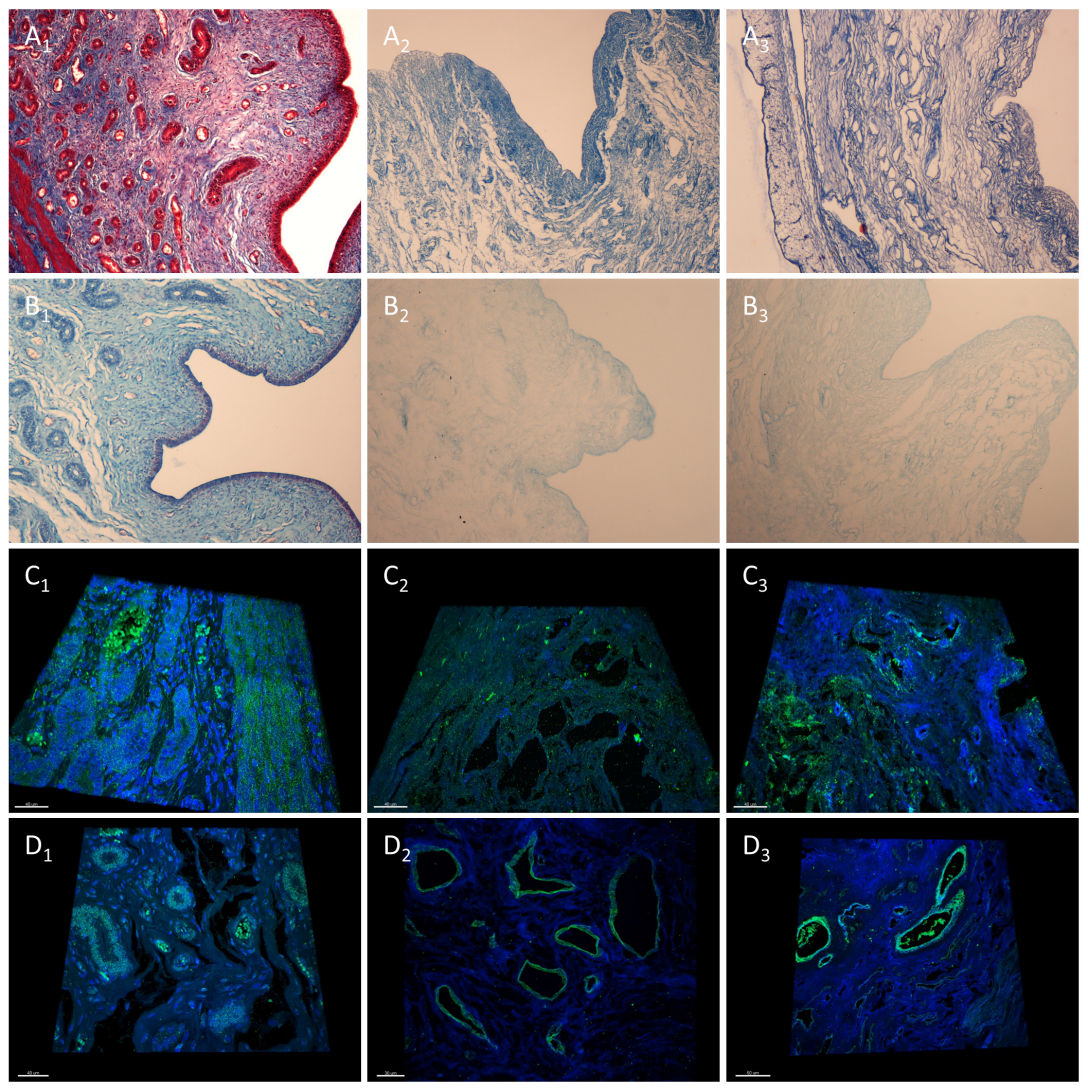
The decellularization of the pig uterus. Representative pictures showing Masson’s trichrome (A1-3) and Alcian blue (B1-3) staining to assess decellularization and the detection of collagen and sulphated GAGs, respectively (10x magnification). Immunofluo-rescence images showing blue signal for nucleus (DAPI) and green signal for the structural ECM protein collagen I (C1-3) and the cell-interacting ECM protein laminin (D1-3), Z-stack over 4 μm (40x magnification). The pig uterus before DC was used as positive control (A1-D1) and compared with the F (A2-D2) and F/T protocol (A3-D3).

DC efficiency was tested first by hematoxylin and eosin (H&E) staining, mounting media containing DAPI was utilized for the detection of nuclear DNA. Quantification of DNA and total protein fraction of untreated and decellularized organs was also performed (Campo et al., 2017). Masson’s Trichrome and Alcian blue staining was used to further confirm the complete DC and analyze the ECM. Both F and F/T protocols resulted in successful DC: H&E and Masson’s trichrome staining showed the complete removal of cellular material and nuclei in all histological layers (peri-, myo-, and endometrium) while preserving the ECM architecture consisting predominantly out of collagens (Figure 2A1-3). The qualitative analysis of sulphated GAGs by Alcian blue staining showed a widespread distribution with a higher signal at the epithelial layer of the endometrium and secretory glands. After DC, only this widespread distribution remained in both protocols with a noticeably lower signal at the epithelium (Figure 2B1-3).

The composition and integrity of the ECM in control and decellularized samples was further analyzed by performing immunofluorescence of five highly constitutive ECM proteins, these are fibronectin, elastin, collagens type I and IV and laminin. For this analysis confocal microscopy was used, this also permitted the creation of 4μm thick 3D image of the samples (supplementary videos 1-6) (scan QR 1-6). Figure 2C and 2D show representative 3D images for collagen I and Laminin, an important structural protein and one vital for cell-ECM interaction respectively. Collagen I, the most abundant collagen remains evenly dispersed in ECM before and after DC (Figure 2C1-3). Laminin is a prominent component of the basement membrane and detected before and after DC in endothelial layers around blood vessels and glandular structures in both layers (Figure 2D1-3). This is also the case for collagen IV, another basement membrane protein, where a signal also observed in the myometrium. Elastin, another major structural constituent of the ECM, was found mainly in the myometrium, basal endometrium, and around arteries. After DC there was a noticeably weaker signal in the interstitial endometrium, while myometrium and arteries remained unaffected. Fibronectin was unaffected and evenly dispersed in ECM, before and after both protocols (Campo et al., 2017). The complete destruction of the nuclei it is also shown by DAPI counterstaining, the remaining DNA signal after DC can be only be found in the tissue fibers. This removal of cellular material was corroborated by DNA and protein quantification: in the pig model the quantification of residual DNA showed a 90.94% and 97.35% drop for the F/T and F protocol, respectively. Quantification of remaining protein fraction showing a 61.02% and 70.03% decrease (Campo et al., 2017).

**Figure 3:**
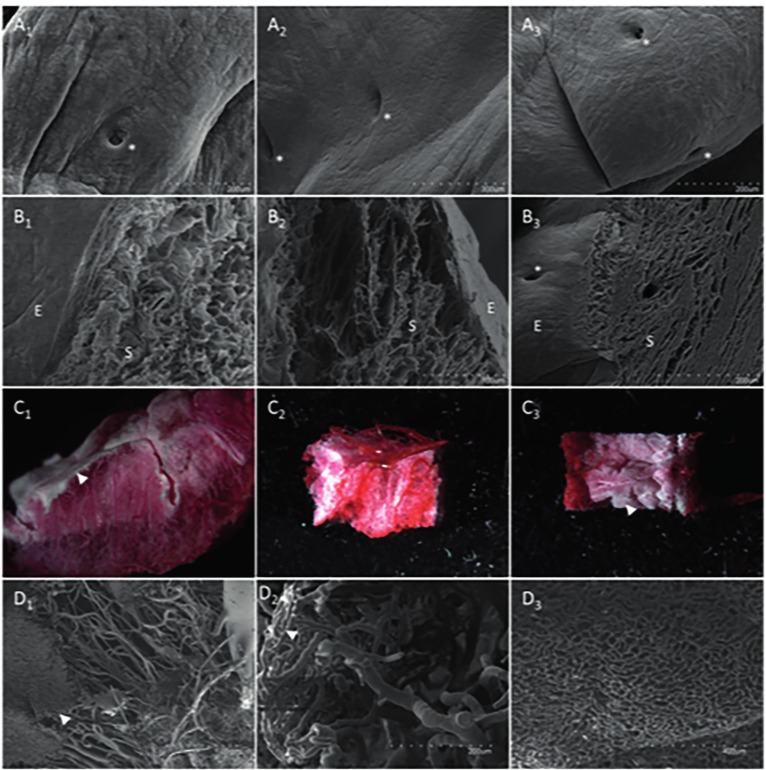
Ultrastructural analysis of endometrial surface, lumen and vasculature after decellularization. Scanning electron micrographs at lower magnification showing the intact lumen of the endometrium (A1-3) and the epithelial (E) and stromal (S) fraction at the E-S interface of the luminal epithelium (B1-3) (300x magnification). Glandular structures are indicated by white asterisks. Sections of the vascular corrosion cast were used for stereoscopic close-up (C1-3) and subsequently for scanning electron micrograph of capillaries (D1-3). White arrowheads show the subepithelial capillary plexus. The pig uterus before DC was used as positive control (A1-D1) and compared with the F (A2-D2) and F/T protocol (A3-D3).

The ultrastructure of control and decellularized samples were investigated by scanning and transmission electron microscopy (SEM and TEM respectively). To assess the integrity of the vascular tree, Batson’s No.17 plastic replica and corrosion kit was used. This created a vascular mold; circular cuts were made, were photographed under a stereomicroscope and submitted to Au-Pd sputtering for SEM. Scanning electron micrographs at low magnification showed that the elements of the endometrial lumen, the epithelial surface and glandular structures remained intact and there were no significant differences observed between all conditions, both before and after DC (Figure 3A1-3 & B1-3). At higher magnification it was clear that the fibers and topography retained their appearance in both protocols and vascular conduits kept their conformation. Transmission electron micrographs showed collagen fibrils, which maintained their striated patterns and were abundant in all orientations throughout the tissue. No noticeable differences at ultrastructural level between the protocols were observed (Campo et al., 2017). The vasculature was investigated by using a vascular corrosion cast, after both DC protocols decreased vasculature integrity was observed macroscopically and under higher magnification. This is likely due to the protocol that perfuses a viscous monomer solution. A region corresponding to the subepithelial capillary plexus was found in all instances, when investigated using SEM, capillaries having the correct thickness were found in all cases (Figure 3C1-3 & D1-3).

#### 1.2 Recellularization of uterine extracellular matrix disks

Finally, to test the in vitro biocompatibility for possible subsequent recellularization, human endometrial Side Population stem cell lines from epithelial (ICE6) and stromal (ICE7) origin (Cervelló et al., 2011) were used to recellularize endometrial disks. For this, tissue from the F protocol were used as it provided the most uniform ECM. Biopsies were oriented with the endometrium facing up and embedded in O.C.T. compound, 100 μm cuts were made with the cryotome. From here 5 mm punch biopsies were made, washed and sterilized. These disks were covered by a viscous pellet containing 0.5 million 4/5 ICE6 and 1/5 ICE7 stem cell mixture and incubated in hypoxic conditions. Endometrial culture medium was changed after 3, 6, and 9 days of culture. After this, the organoid-like structure was embedded in paraffin and serial cuts were made. Successful recellularization (RC) and correct cell differentiation were assessed by H&E staining and immunofluorescence against human vimentin and cytokeratin.

**Figure 4:**
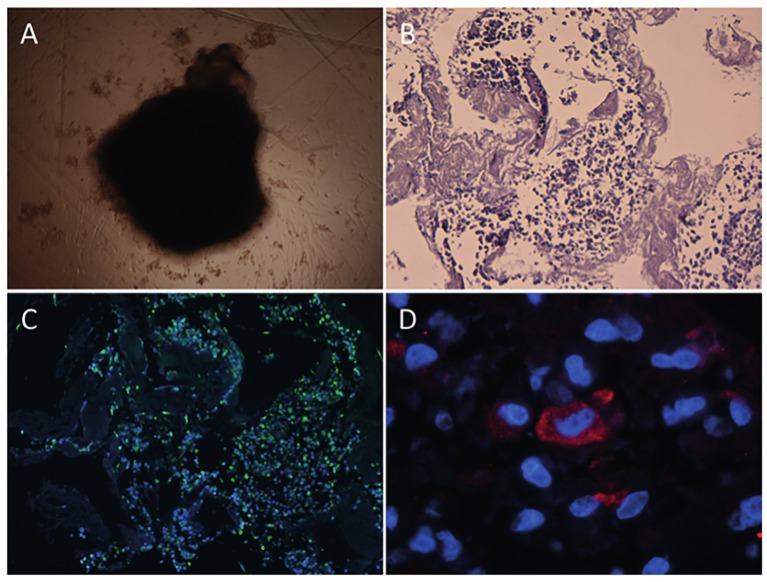
Organoid-like structure formation, histology and immunofluorescence analysis. Endometrial decellularized discs formed an organoid-like structure after 3-4 days under hypoxic culture conditions (A, scale bar = 50 μm). H&E staining showing the close interaction of the cells with the ECM after cell seeding and 9-12 days of culture on scaffold (B, 10x magnification). Immunofluorescence images of vimentin (green) positive cells, nuclear staining appears blue (DAPI) (C, 10x magnification). Detail of cytokeratin (red) positive cell (D, 100x magnification).

After seeding human SP cells, the coated scaffolds rolled up and contracted, forming an organoid-like structure (Figure 4A). H&E staining showed that the cells were encapsulated within the decellularized scaffold, demonstrating that cells were attaching and interacting closely with the endometrial ECM (Figure 4B). To demonstrate the tissue-specific phenotypical behavior of the SP stem cells, immunofluorescent staining of typical endometrial markers was performed. Cells with a positive signal for vimentin, an intermediate filament protein expressed in mesenchymal cells, were observed throughout the entire structure (Figure 4C). Cells expressing cytokeratin, a marker for epithelial cells, were also present in the organoid-like structure (Figure 4D).

### 2. The rabbit model: decellularization of whole uterus, solubilization of acellular endometrium and embryo culture on tissue-engineered substrata.

#### 

The goal of this study was to create coatings and hydrogels from decellularized endometrial tissues in different phases of proliferation and compare the in vitro embryo culture development on these substrata, on Matrigel coating and hydrogels and standard culture conditions. Two types rabbit uteri were used: or the were obtained from rabbits that were not stimulated or they came from rabbits 72 hours after ovarian stimulation. The latter type will be referred to as “synchronous” (S) from here on because of the synchronization between the embryo and tissue (both at day 3).

In total 8 different embryo culture conditions were tested: on top of three different biological surface coatings (C) and hydrogels (H) made from non-synchronous (NSC and NSH) acellular endometrium, synchronous (SC and SH) acellular endometrium and Matrigel (MC and MH) and in two standard culture conditions using uncoated wells with culture medium supplemented with and without 10% Fetal Bovine Serum (FBS), C+FBS and C-FBS respectively. After that, hatching rates, morphometry and expression of 3 core pluripotency markers were analyzed and compared.

#### 2.1 Whole uterus decellularization

Four whole uteri were first decellularized, 2 of which were non-synchronous and 2 from synchronous rabbits. The pig DC protocol was adapted (Table II), the very last 5 hrs of PBS perfusion were replaced by 1 hour of PBs, 1 hour of 2μg/mL DNase 1 solution and 3 hours of PBS. The perfusion speed was set at 8 mL/min per horn. The decellularization efficiency was assessed using H&E, MT and DAPI staining, followed by DNA and protein quantification. These histological techniques showed similar results as presented in the pig model. Furthermore, a 95.3% and 93.2% reduction in double stranded DNA for the non-synchronous and synchronous decellularized uterus respectively were measured while protein concentration showed a 45.9% and 38.2% decrease in in both tissues respectively (Campo et al., 2019).

#### 2.2 Preparation and properties of non-synchronous and synchronous ECM hydrogel

Microdissection was able to obtain pure endometrial tissue in both control and decellularized tissues. This NS and S acellular endometrium was lyophilized and milled with dry ice in an ultracentrifugal mill to reach grain size smaller than 0,0625mm^2^. This powder was digested in pepsin until no more tissue was visible. When left at 37°C for one hour, the resulting viscous pre-gel solutions formed stable hydrogels that required gentle handling. Long-term structural stability and sterility of this ECM-derived gel was demonstrated by incubating them at 37°C in standard cell culture medium for 7 day. The hydrogel remained intact during this period and no bacterial growth was observed.

**Figure 5:**
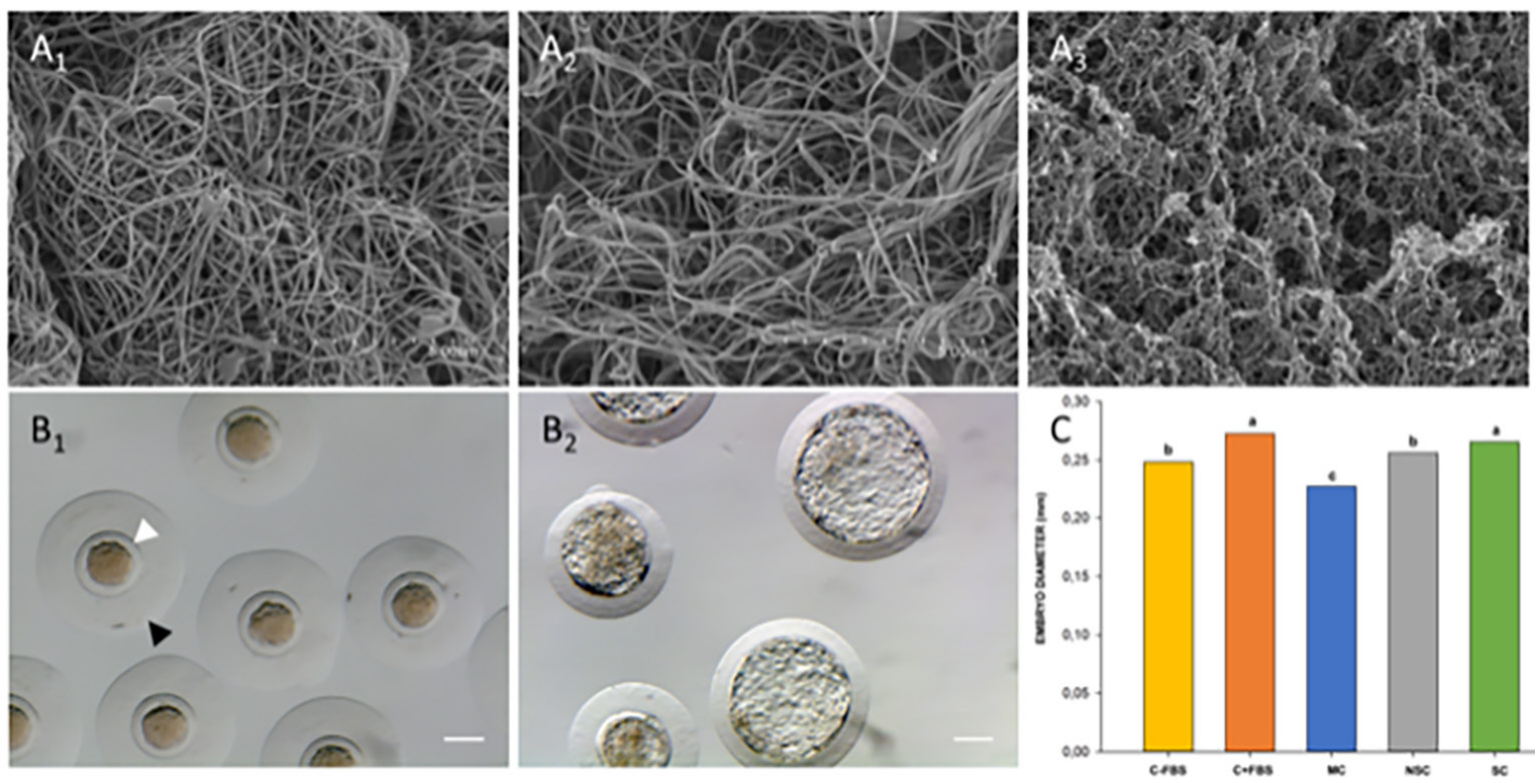
Characterization and biological effect of ECM substrates. Representative scanning electron micrographs of non-synchronous gel (A1), synchronous gel (A2) and Matrigel (A3). ECM hydrogels have 50 - 150 nm thick nanofibers and show similar density, scale bars are 5 μm. Late stage morulae/early stage blastocyst after collection showing homogenous cellular mass, spherical zona pellucida (white arrowhead) and mucin coat (black arrowhead) (B1) and fully hatched blastocyst after 48hrs of in vitro culture (B2, scale bars are 0.1mm). Comparison of mean hatched embryo diameter values (C, adapted from (Campo et al. 2019)). a–c: Data with uncommon letters are different. Differences were considered if the probability of the difference between groups was ≥0.8 (80%) using Bayesian inference methods.

These hydrogels were further characterized, to analyze the fiber density and thickness, scanning electron microscopy (SEM) was used and fiber diameter was measured using ImageJ software. Scanning electron microscopy of the gel surface showed that both NS and S gels have a homogenous, randomly interlocking fibrillar structure of similar density (Figure 5 A1-3). Fiber diameter of the NS and S hydrogels was analyzed from images taken at 60.000X resolution (>30 measurements per gel). The diameter of the re-assembled nanofibers from both gels range between 50 and 149 nm, measuring on average 70.04 ± 9.15 vs 102.90 ± 22.66 for the non-synchronous and synchronous hydrogel respectively. A significant difference between both fiber diameters was found.

The gelation kinetics were evaluated by turbidimetry, 100μl of each solution was added in triplicate in 96-well plates at 37°C and measured spectrophotometrically in a plate reader. Absorbance was measured at 405 nm every 2 min and values were normalized. Two gelation kinetic parameters were calculated: the time to half gelation (t1/2) was defined as the time necessary to reach 50% of the maximal absorbance, and the lag time (tlag) was defined as the moment that the gelation curve increases from 0% normalized absorbance. Gelation kinetics of both gels followed a sigmoidal curve, but significant differences were observed in the parameters. Gelation of the S hydrogel started almost immediately and tended to have a lower lag time than the NS hydrogel (tlag =2.98 ± 0.86 min vs 4.9 ± 0.33 min). The time to reach 50% of the maximal gelation did show significant differences, where the S gel needed about 5 minutes less than the NS hydrogel (t1/2 = 10.43 ± 0,74 min and 15.46 ± 0,29 min, respectively) (Campo et al., 2019).

#### 2.3 Biological characteristics and applications of hydrogels

As described earlier, day 3 late stage morulae were cultivated for 48 hours in 8 conditions comparing synchronous, non-synchronous and Matrigel substrata vs control conditions. The in vitro development ability of embryos was assessed based on the hatching/hatched rate (proportion of hatching and hatched blastocyst at 48h of culture from total cultured embryos). In vitro development was significantly affected by the hydrogel culture conditions, averaging around 27.64±26.38%. However, this was not the case for the coating conditions, the mean value of hatching rate here was 89.4±3.13% (Table III).

**Table III:** — Hatching rates of day 3 embryos after 48 hours of incubation on different culture conditions.

Hatching state	NSC	SC	MC	C-FBS	C+FBS	NSH	SH	MSH
Stopped	21	10	22	3	1	66	55	28
Hatched/hatching	70	82	64	74	58	4	14	37
Total amount	91	92	86	77	59	70	69	65
Ratio hatched /hatching embryos	76,92 %	89,13 %	74,42 %	96,10 %	98,31 %	5,71 %	20,29 %	56,92 %

Abbreviations: FBS: fetal bovine serum; MC: Matrigel coating; MH: Matrigel hydrogel; NSC: non-synchronous coating; NSH: non-synchronous hydrogel; SC: synchronous coating; SH: synchronous hydrogel.

Following this, a morphometric analysis of the hatched embryos was performed and the gene expression profile of the three core pluripotency factors (OCT4, NANOG and SOX2) was assessed. This was only done with the control (C-FBS and C+FBS) and coating embryo culture batches (NSC, SC, MC) because of the vastly inferior development in the hydrogel groups. Differences between the experimental groups were estimated using Bayesian inference. Both descriptive statistics and phenotypic differences were computed with the program Rabbit developed by the Institute for Animal Science and Technology (Valencia, Spain), a more detailed description can be found in (Blasco, 2005).

For the morphometric analysis, the diameters of the hatched embryos were measured and compared. The hatched state was achieved when more than 50% of the embryonic mass cell was extruded of the zona pellucida, figure 5B1 and B2 show the correct embryo development on substrata. The mean diameter of the embryos of SC and C+FBS groups was significantly higher than the diameter of the NSC, C-FBS and MC groups after 48 hours of in vitro culture (Figure 5C). In addition, the patter of mRNA expression of NANOG was not significantly affected by culture conditions. Nevertheless, blastocysts developed in NSC group showed higher levels of OCT4 transcript abundance than C-FBS group, while SOX2 transcript abundance was higher in the blastocysts developed in SC, NSC and C+FBS groups, in comparison with the MC and C-FBS groups (Campo et al., 2019).

## Discussion

With more than 70 million couples affected, infertility is considered a worldwide public health issue, affecting both males and females, many of which come from developed countries (Ombelet et al., 2008). In the United States alone, 12% of 15-44 years old women (7.3 million) have impaired fecundity (data from 2002) (Chandra et al., 2005). These issues resulted in the birth of medically assisted reproduction, and great advances in treating pathologies leading to reproductive dysfunction have been made ever since. However, some of the diseases resulting in female infertility are still poorly treatable and can profit greatly from advances made by reproductive bioengineering.

One of the main afflictions in reproductive medicine that was untreatable until recently is Absolute Uterine Factor Infertility (AUFI). gestational surrogacy is the only alternative to conceive genetically related offspring; however, it is an expensive solution with many legal and ethical challenges/problems (Ber, 2000; Shenfield et al., 2005). The only definitive treatment for AUFI is allogeneic transplantation and is now becoming a true option, as demonstrated by the Brännström group (Brännström et al., 2015). However, apart from the inherent risk of surgery, there are long waiting periods caused by the lack of compatible donor organs and the need for long-term immunosuppression after the transplantation. The latter is associated with severe side effects lowering the quality of life of the patients. Examples of this are nephrotoxicity, increased susceptibility to infections, diabetes and accelerated arteriosclerosis (Azimzadeh et al., 2011).

Many techniques and technologies are being developed to resolve the problems affecting organ transplantations. One line of investigation, the de- and recellularization of whole organs, has shown promising results to possibly someday engineer transplantable organs. Here, organs could be regenerated by incubating vascularized complex scaffolds with cells in a bioreactor to create, in theory, an endless supply of allogeneic donor organs that do not trigger an immune response (O’Brien, 2011). The DC procedure’s success depends on tissue density, thickness, and cellularity. Hence, protocols must be adapted to the organ of interest; one technique does not always translate from one organ to the other or even between the same organs of different species, and optimization and comparison of protocols is necessary (Hellström et al., 2014; Santoso et al., 2014). Because of this, many specialized DC protocols are established in a multitude of organs (Ott et al., 2008; Crapo et al., 2011; Sullivan et al., 2012; Baptista et al., 2013).

In the first part of this thesis, a comparison was made between F/T versus fresh pig uteri, using a protocol consisting of SDS and Triton X-100 cycles. After two identical 24-hr cycles a macroscopically acellular matrix was produced, having a semi-transparent and white appearance. SDS is an ionic detergent that is widely used for DC, when used in low concentrations it can effectively remove cell residues with minimal damage to the ECM (Crapo et al., 2011). Furthermore, there was little to no effect of the prior F/T step on the ECM architecture in our experience. This is encouraging, since a single F/T cycle can reduce adverse immune response, making it an convenient and interesting first step for both preserving and decellularizing donor organs (Lehr et al., 2011).

The absence of nuclear and cytoplasmatic components allows us to consider the organ as a “decellularized scaffold” (Crapo et al., 2011). Additionally, the physical and possible mechanical properties of ECM were demonstrated by the presence of native collagen fibers visualized with Masson’s trichrome and Alcian blue staining. The major components of the ECM, such as collagens I and IV, elastin, laminin and fibronectin, were preserved. These results and those of electron microscopy imply that the interstitial ECM proteins and basement membrane remained intact under both DC conditions. These key components act in the microenvironment to provoke changes like cell migration, proliferation, and differentiation (Halper and Kjaer, 2014). An intact vasculature system is also important, first for the perfusion-DC but also for the future repopulation of the organ (Sánchez et al., 2015). It was demonstrated that protocols only slightly compromised the vascular integrity, showing perfusable vascular conduits up to the capillary level which opens the possibility to recellularize the whole organ by adding cells via perfusion or injection and create a tissue-engineered neo-organ (Ott et al., 2008; Miyazaki and Maruyama, 2014).

The final step was to verify the biocompatibility and bio-inductive properties of thin sections of the generated xenogeneic endometrial scaffolds in vitro. After 3 to 4 days the seeded scaffolds contracted and rolled up and 7 days later organoid-like structures were observed. Histology showed a tight interaction between the cells and the scaffold. Furthermore, endometrial cells of both epithelial and stromal fractions were identified in the structure.

For the second part, we demonstrated the successful DC of whole rabbit uteri and the isolation of acellular endometrial tissue. The endometrium suffers, under the influence of the female sex steroids, cycles of drastic growth and reorganization in order to accept the embryo (Jabbour et al., 2006; Evans et al., 2011). For the rabbit, these changes already start from the first days post coitum (Denker, 1982). The ECM of the endometrial tissue follows this cycle with differential expression of both interstitial and basement membrane proteins, many of which remain after DC (Yamanaka et al., 1996; Harrington et al., 1999; Jones-Paris et al., 2017). The endometrial cells produce different secretory compounds and growth factors during the different phases of the endometrial cycle (Parmar et al., 2008). It is possible that these could be sequestered by the native ECM and retained after DC (Ishihara et al., 2018). The decellularization and solubilization of tissues has been rising in popularity and has been applied to all important organ systems (Saldin et al., 2017). To the best of our knowledge, this has never been done before for reproductive tissues until now (Campo et al., 2019).

When comparing the ultrastructure of the NS and S hydrogel, significantly thicker fibers are observed. However, the development of the embryos was severely impaired when they were cultured on these NS, S or M hydrogels, in contrast to their coating counterparts. One possible explanation could be that the hydrogel density and rigidity play a role in the development. An interesting future experiment could use coated polyacrylamide gels with various degrees of stiffness elucidate its effect, similar experiments have been performed to recreate the biomechanical cues of the cell niche (Young et al., 2013).

Rabbit blastocyst use a fusion type of implantation and does not invade the endometrium, nonetheless, it has been reported that it also produces proteases (Denker, 1982). It is possible that these proteases produced by the blastomeres of hatching embryos release more proteins from the substratum, positively influencing embryo development. When the coating was made from the synchronous endometrium, the positive effect yielded blastocysts with hatching rates, size resembling those grown in standard positive conditions, effectively compensating for the absence of adhesion proteins and growth factors of the serum in the culture medium.

A similar pattern of pluripotency markers OCT4 and SOX2 was seen in the synchronous endometrium and serum group. They are part of the Sox2-Oct4-Nanog regulatory complex, which controls expression of pluripotency genes through feed-forward loops including these three genes in an autoregulatory circuit (Chickarmane et al., 2006; Okumura-Nakanishi et al., 2005). It is possible that an inadequate mRNA expression plays a vital role in development and implantation of the embryos, the important role of Sox2 in the preimplantation mouse embryo further indicates this (Keramari et al. 2010). Taken all together, it appears that the S coating was able to sequester and release biomimetic compounds under in vitro conditions.

## Conclusions

In this thesis, we intended to develop tissue-engineering approaches based on decellularized uterine tissues obtained from whole organs to improve several aspects within reproductive medicine. We hypothesized that the decellularization of whole uteri from different species has not only the potential to, one day, create tissue-engineered, transplantable organs as but that the DC endometrial fraction can also be processed further into thin sections, ECM hydrogels and coatings that can be used as a biocompatible tissue-specific substrates for cell and embryo culture. Furthermore, endometrial ECM hydrogels and coatings have the potential to become a platform used in the culture of stem/progenitor cells and primary culture cells to better maintain their tissue-specific phenotype, improving in vitro models. This can also have in vivo applications, such as the treatment of Asherman’s syndrome and endometrial atrophy.

## Video scan (read QR)

Supplementary video 1: Three-dimensional Z-stack showing immunofluorescence signal of collagen I in por-cine fresh uterus control (40x magnification).

Supplementary video 2: Three-dimensional Z-stack showing immunofluorescence signal of collagen I in F-protocol decellularized uterine tissue (40x magnification).

Supplementary video 3: Three-dimensional Z-stack showing immunofluorescence signal of collagen I in F/T-protocol decellularized uterine tissue (40x magnification).

Supplementary video 4: Three-dimensional Z-stack showing immunofluorescence signal of laminin in por-cine fresh uterus control (40x magnification).

Supplementary video 5: Three-dimensional Z-stack showing immunofluorescence signal of Laminin in F-pro-tocol decellularized uterine tissue (40x magnification).

Supplementary video 6: Three-dimensional Z-stack showing immunofluorescence signal of Laminin in F/T-protocol decellularized uterine tissue (40x magnification).

**Figure:**